# Severity Biomarkers in Puumala Hantavirus Infection

**DOI:** 10.3390/v14010045

**Published:** 2021-12-28

**Authors:** Tuula K. Outinen, Satu Mäkelä, Ilkka Pörsti, Antti Vaheri, Jukka Mustonen

**Affiliations:** 1 Department of Internal Medicine, Tampere University Hospital, Elämänaukio 2, 33520 Tampere, Finland; satu.m.makela@pshp.fi (S.M.); ilkka.porsti@tuni.fi (I.P.); jukka.mustonen@tuni.fi (J.M.); 2 Faculty of Medicine and Health Technology, Arvo Ylpön katu 34, Tampere University, 33014 Tampere, Finland; 3 Department of Virology, Medicum, University of Helsinki, 00290 Helsinki, Finland; antti.vaheri@helsinki.fi

**Keywords:** hantavirus, hemorrhagic fever with renal syndrome, Puumala virus, biomarker

## Abstract

Annually, over 10,000 cases of hemorrhagic fever with renal syndrome (HFRS) are diagnosed in Europe. Puumala hantavirus (PUUV) causes most of the European HFRS cases. PUUV causes usually a relatively mild disease, which is rarely fatal. However, the severity of the infection varies greatly, and factors affecting the severity are mostly unrevealed. Host genes are known to have an effect. The typical clinical features in PUUV infection include acute kidney injury, thrombocytopenia, and increased vascular permeability. The primary target of hantavirus is the endothelium of the vessels of different organs. Although PUUV does not cause direct cytopathology of the endothelial cells, remarkable changes in both the barrier function of the endothelium and the function of the infected endothelial cells occur. Host immune or inflammatory mechanisms are probably important in the development of the capillary leakage. Several immunoinflammatory biomarkers have been studied in the context of assessing the severity of HFRS caused by PUUV. Most of them are not used in clinical practice, but the increasing knowledge about the biomarkers has elucidated the pathogenesis of PUUV infection.

## 1. Introduction

Hantaviruses are enveloped viruses with a trisegmented viral RNA genome [1]. The segments called small, medium, and large encode the nucleocapsid protein N, the two glycoproteins Gn and Gc, and the RNA polymerase, respectively [1]. Hantaviruses can cause two distinct syndromes in humans, i.e., hemorrhagic fever with renal syndrome (HFRS) in Europe and Asia, and hantavirus cardiopulmonary syndrome (HCPS) in the Americas [1,2,3]. The Hantaan virus (HTNV), Puumala virus (PUUV) and Dobrava–Belgrade virus (DOBV) cause HFRS in Eurasia. The Seoul virus (SEOV) causing HFRS is a global pathogen [1,2,3]. Over 10,000 HFRS cases are diagnosed annually in Europe [2]. PUUV, carried by the bank vole (*Myodes glareolus*), causes most of the European HFRS cases [4]. The majority of these infections are reported in Finland, which has the highest incidence globally of a diagnosed hantavirus disease, with 1000–3000 serological diagnoses each year [4,5].

PUUV infection is typically associated with increased vascular permeability, acute kidney injury (AKI), and thrombocytopenia [6,7,8,9,10]. Whereas the case fatality rates for HFRS caused by Dobrava–Belgrade virus are up to 14%, the lethality of PUUV infection is low, 0.1–0.4% [2,5,11]. However, the disease often leads to hospitalization and sometimes, intensive care unit treatment, including renal replacement therapy, is needed [9]. Furthermore, several long-term nephrological, cardiovascular, and endocrinological consequences have been described after PUUV infection [2]. Factors affecting the severity of PUUV infection remain mostly unclear. However, host genes are known to influence the outcome [12,13,14,15]. No vaccine or specific therapy is at present available for the disease.

A central phenomenon in hantavirus infections is increased capillary permeability, leading to vascular leakage, edema in various tissues, and decreased blood pressure [1,9]. The detailed pathogenetic mechanisms underlying this phenomenon are unclear. Hantaviruses replicate predominantly in endothelial cells of the capillaries of various organs, and the endothelium is the primary target of a hantavirus infection [1]. However, PUUV does not cause direct cytopathology or apoptosis of endothelial cells, but nonetheless, the infection leads to remarkable changes in both the barrier function of the endothelium and the function of the infected endothelial cells [1,9]. Therefore, immunological or inflammatory host mechanisms are probably important in the pathogenesis of the capillary injury [1,9]. Generally, PUUV induces an intense immune response that involves both B and T cells [16]. Cytotoxic T cells may trigger the increased capillary permeability, while cytokines and complement activation contribute to the progression of the capillary leakage [1].

Several biomarkers have been studied in PUUV infection to have a better comprehension of the diversity of the clinical picture and to assess the outcome of the infection. In this review, we summarize the current knowledge about different biomarkers in PUUV hantavirus infection.

## 2. Biomarkers in PUUV Infection

### 2.1. Interleukin-6

Interleukin-6 (IL-6) is a multifunctional cytokine involved in immune responses and inflammation [17,18]. It is produced by various types of cells, such as monocytes, macrophages, lymphocytes, endothelial cells, and fibroblasts, and it plays a pivotal role during the transition from innate to acquired immunity [17,18]. IL-6 induces the production of acute phase response proteins by the liver and expands the inflammatory responses by activating T cells and promoting the proliferation and differentiation of B cells [17]. Although IL-6 is mostly regarded as a pro-inflammatory cytokine with potentially harmful effects, it has also many regenerative or anti-inflammatory functions due to the stimulated synthesis of anti-inflammatory cytokines and subsequent limitation of inflammation [18]. In addition to being involved in microbial immune responses, a high production of IL-6 has been detected in several acute and chronic conditions, such as various autoimmune diseases, AKI, acute myocardial infarction, and critical illness, and it has been shown to predict morbidity and mortality in many of these conditions [19,20,21,22]. Furthermore, in numerous bacterial and viral infections, including septicemia, pneumonia, influenza, and HIV infection, high IL-6 has predicted worse outcome [23,24,25,26]. In Crimean-Congo hemorrhagic fever and in HCPS, IL-6 has predicted severity of the clinical outcome as well as risk of death [27,28,29]. As the hallmark in hantavirus infections is the capillary leakage caused by endothelial dysfunction, it is of interest that IL-6 has been shown to cause endothelial barrier dysfunction via the protein kinase C pathway [30]. IL-6 together with other pro-inflammatory cytokines probably plays an important role in the development of the vascular leakage.

In PUUV-infected patients, IL-6 production is increased, and high plasma levels are associated with a more severe clinical disease [28,31,32,33,34,35]. High plasma IL-6 associates with more severe thrombocytopenia and leukocytosis as well as longer stay in hospital [33,34]. Moreover, high plasma IL-6 predicts severe AKI in PUUV infection [32,33]. Furthermore, urinary IL-6 excretion is increased during acute PUUV infection, and it correlates with the amount of albuminuria [35]. However, urinary excretion of IL-6 does not correlate with plasma IL-6 concentration, indicating probable local production of IL-6 in the kidneys [35]. Overall, IL-6 appears as a useful biomarker when assessing the severity of PUUV hantavirus infection.

### 2.2. C-Reactive Protein

CRP was originally described and named for its ability to bind in a calcium-dependent way the C-polysaccharide of *Streptococcus pneumoniae* [36]. CRP is the prototype of an acute phase response protein produced in the liver during various inflammatory and infectious conditions mainly in response to IL-6 [37]. The main functions of CRP are the activation of the classical complement pathway, induction of cytokine production, as well as enhancement of phagocytosis, and thus, CRP serves as an opsonin for infectious agents and damaged cells [37,38]. CRP plays an important role in the innate immune response against different micro-organisms, and it is rapidly increased up to 1000-fold after the onset of a stimulus [38]. Numerous conditions in addition to infections and inflammation, such as trauma, malignancies, and myocardial infarction can stimulate the production of CRP, and it may also have a role in the development of atherosclerosis and cardiovascular diseases [37,38]. CRP is widely used in clinical practice in the evaluation of disease severity during different infections as well as inflammatory disorders, and it is also used to discriminate between bacterial and viral infections, although the concentrations partly overlap [39]. The higher concentrations in bacterial than viral infections result from different inflammatory profiles. Many bacterial diseases are characterized by elevated levels of circulating IL-1β and IL-6 with a concomitant increase in plasma CRP, while viral infections are commonly characterized by elevated levels of the pro-inflammatory cytokine IL-18 [40]. In addition to being an antimicrobial molecule and a marker of inflammation, CRP protects against autoimmunity by binding autoantigens and promoting the clearance of apoptotic cells [41]. Furthermore, besides activating the classical pathway of complement, CRP also regulates excess inflammation by inhibiting the alternative and terminal complement pathways [42].

In PUUV infection, almost all patients have elevated CRP levels [6,7,33]. However, the maximum CRP levels vary widely. In a Finnish study, the maximum level of CRP ranged 11–269 mg/L, and the median level was 69 mg/L [33]. Studies have found controversial results regarding the ability of CRP to predict the severity of the disease. In the Finnish study, CRP did not associate with a more severe disease in terms of the severity of thrombocytopenia, leukocytosis, or AKI, nor the length of hospital stay in PUUV-infected patients [33]. Conversely, CRP appeared as a protective factor against severe AKI in this study [33]. High CRP could help in preserving kidney function by reducing the deposition and increasing the clearance of immune complexes in the renal cortex [33]. However, a German study found an association of high CRP with a more severe AKI [43]. Thus, the predictive and possible protective role of CRP in PUUV infection remains to be elucidated.

### 2.3. Pentraxin-3

Pentraxin-3 (PTX3) acts as a soluble pattern recognition receptor participating in the innate host defense against certain pathogens [44]. It is an acute phase protein generated at the site of inflammation in various cells and tissues, mainly by dendritic cells, neutrophils, mononuclear phagocytes, vascular endothelial and smooth muscle cells, and fibroblasts in response to inflammatory signals [44]. In addition to mediating inflammatory activation, PTX3 can act as a negative feedback regulator, limiting excessive neutrophil recruitment [45]. PTX3 recognizes different pathogens, bacteria, viruses, and fungi, modulates complement activity by binding C1q, and facilitates pathogen recognition by macrophages and dendritic cells [46]. Furthermore, PTX3 interacts with factor H, which activates the alternative pathway of the complement system [47]. Interestingly, PTX3 has been demonstrated to possess direct antiviral activity against cytomegalovirus and influenza virus [48,49]. Moreover, PTX3 has a protective role in murine hepatitis virus-induced acute lung injury and in mouse models of myocardial infarction and atherosclerosis [50,51,52]. In patients with HFRS caused by HTNV, PTX3 has demonstrated significant predictive value for the mortality risk [53].

In PUUV infection, plasma PTX3 levels are elevated during the acute phase [54]. High PTX3 level associates with a clinically severe course of the disease, especially severe AKI, thrombocytopenia, and longer hospitalization [54]. Most of all, a high PTX3 level predicts significant thrombocytopenia. The PTX3 level also correlates with the activation of the complement system in PUUV infection [54]. Plasma terminal complement complex SC5b-9 levels, in turn, correlate with severe thrombocytopenia in infection by PUUV [55]. Thus, PTX-3 is thought to have a role in the disease pathogenesis due to the cross-linkage of coagulation and complement system activation [9,56,57].

### 2.4. Indoleamine 2,3-Dioxygenase

Indoleamine 2,3-dioxygenase (IDO) is an interferon-induced enzyme having a rate-limiting role in the catabolic pathway of the essential amino acid tryptophan [58]. It is produced by dendritic cells, macrophages, B cells, and endothelial cells, as well as several other types of cells, including renal tubular epithelial cells [58,59]. IDO-mediated tryptophan depletion has an inhibitory influence on cellular metabolism, and thus, IDO has predominantly immunosuppressive effects, with T cells being particularly affected [60]. In addition, tryptophan degradation products promote the generation and activity of regulatory T cells, and thus, IDO limits tissue inflammation and autoimmunity [58]. Equally, IDO can reduce excessive immune reactivity to chronic infections and cancer [61]. In infectious diseases, IDO has a dualistic role. It can directly attenuate bacterial replication or the progression of a viral infection, but it can also suppress the necessary host immune responses [9]. These immunoregulatory functions of IDO have been linked to negative outcomes in several chronic or latent viral infections [62].

In acute PUUV infection, serum IDO levels are elevated [63]. High IDO levels are also associated with increased disease severity [63]. Most of all, a high IDO level is associated with a more severe AKI, more pronounced inflammation, and longer hospital stay in acute PUUV infection [63]. IDO might not merely be a marker of the severity of PUUV infection, but it could be involved in the pathogenesis by inducing immunosuppression and thus enhancing the infection [63]. In PUUV-infected patients, IDO activity has been found to be linked to the suppressive capability of regulatory T cells but not to the proliferative responses of effector T cells [62]. This suggests that the mechanism responsible for the suppressive effect of IDO is not metabolic control of the effector cells but rather the signaling mediated by tryptophan breakdown products [62]. Interestingly, increased IDO activity also promotes tubular epithelial cell apoptosis, and this provides another possible pathogenetic mechanism for IDO in PUUV infection [59].

### 2.5. Cell-Free DNA

Circulating plasma cell-free DNA (cf-DNA), i.e., detectable DNA fragments in plasma, is probably released from apoptotic or necrotic cells, although secretion by living cells has also been proposed as a source of cf-DNA [64]. Increased cf-DNA levels have been detected in various clinical disorders, including traumas, infections, tumors, autoimmune diseases, and sepsis [64,65]. In several of these conditions, the cf-DNA level has also provided good prognostic value to predict future outcomes [64,65]. Elevation of cf-DNA in pathological states is likely due to increased cell death rate rather than immunological activation, which makes cf-DNA a very sensitive but at the same time an unspecific marker of tissue damage [9]. Interestingly, in HFRS caused by HTNV, plasma cf-DNA level was elevated in the early stages of the infection and correlated with HTNV load and disease severity [66]. Moreover, in most patients, qualitative analysis indicated cf-DNA to be of apoptotic origin [66].

In PUUV infection, plasma cf-DNA levels are elevated [67]. Furthermore, during the acute phase of the infection, cf-DNA displays in qualitative analysis a predominance of apoptotic appearance [67]. Thus, high cf-DNA levels probably reflect apoptosis occurring during the acute phase of PUUV infection [67]. The total plasma cf-DNA level also correlates with the severity of the disease in terms of thrombocytopenia, leukocytosis, and the length of hospital stay but not with the severity of AKI [67]. In turn, the urinary excretion of cf-DNA is not increased in acute PUUV infection and does not correlate with the severity of the infection, including the severity of AKI [67]. Therefore, the urinary excretion of cf-DNA does not reflect the degree of inflammation in the kidney [67].

### 2.6. Soluble Urokinase-Type Plasminogen Activator

Urokinase-type plasminogen activator receptor (uPAR) is a multifunctional glycoprotein upregulated during infection and inflammation and involved in many different immune functions [68,69]. It is expressed in several different cells including monocytes, lymphocytes, neutrophils, macrophages, endothelial cells, kidney tubular epithelial cells, as well as podocytes [68,69,70]. The uPAR interacts with several molecules mediating immune system signals and promotes the migration and adhesion of leukocytes by binding to β-integrins [68]. Plasma levels of soluble uPAR (suPAR) reflect the activation level of the immune and inflammatory systems [69]. Plasma suPAR is increased and also has prognostic value in various conditions, such as autoimmune diseases, cancer, and various infections [69,71,72,73]. The suPAR can activate β_3_-integrin within the kidney podocytes, leading to podocyte dysfunction and effacement and proteinuria [74]. Furthermore, suPAR has been shown to induce nephrin down-modulation in human podocytes, which may affect kidney functionality [75].

In PUUV infection, plasma suPAR levels are markedly elevated [76,77]. Moreover, high suPAR level correlates with the severity of the infection, especially thrombocytopenia, leukocytosis, more severe AKI, and longer hospitalization [76]. Serum suPAR concentration has also been found to correlate with proteinuria and urinary nephrin level [77]. Furthermore, the excretion of suPAR into the urine is increased in acute PUUV infection [78]. Urinary suPAR level correlates with the severity of the disease, the length of the hospital stay, the severity of AKI, and the degree of proteinuria [78]. However, urine suPAR does not correlate with plasma suPAR, indicating that high urine suPAR probably reflects local production in the kidney during the acute infection [78].

Taking into account the known effects of suPAR on kidney podocytes and the mechanisms of proteinuria, it is probable that suPAR is not only a biomarker of the severity of the disease but has also a pathogenetic role in the development of proteinuria during PUUV infection.

### 2.7. Resistin

Adipokines that regulate appetite and energy metabolism are bioactive molecules, which were first found to be secreted by adipose tissue. Later, adipokines have been discovered to be produced by many other cell types, particularly by inflammatory cells, and to regulate inflammatory responses [79]. Resistin is an adipokine mainly produced by mononuclear leukocytes and especially macrophages [80]. Plasma resistin changes have been found in acute infections and AKI [81,82,83]. Furthermore, increased resistin concentrations are observed during several inflammatory diseases, including sepsis [84,85]. Plasma resistin levels are significantly higher in patients with septic shock and AKI when compared to patients with septic shock without AKI, and resistin also modulates the inflammatory response in those patients [83]. Moreover, plasma resistin levels are elevated in acute Dengue fever and Crimean-Congo hemorrhagic fever, and plasma resistin levels also associate with severe disease [82,86].

In PUUV infection, plasma resistin concentrations are elevated in the acute phase [87]. High resistin levels correlate with the severity of AKI, as well as with several other markers reflecting the severity of the disease, e.g., thrombocytopenia and the length of hospital stay [87]. High plasma resistin levels during the acute infection also associate with hematuria and albuminuria [87]. The association of resistin with the amount of albuminuria suggests that the plasma resistin level is not only influenced by renal clearance but could have some role in the pathogenesis of AKI during PUUV infection [87].

### 2.8. YKL-40

YKL-40, also known as chitinase 3-like protein 1 (CHI3L1), is a heparin and chitin-binding inflammatory glycoprotein secreted by a variety of cells, particularly by activated macrophages and neutrophils, in different tissues characterized by inflammation [88,89]. It is also produced by vascular smooth muscle cells in response to endothelial damage [89]. YKL-40 has a role in the activation of the innate immune system and maturation of monocytes to macrophages [89]. It is involved in endothelial dysfunction by promoting chemotaxis, cell attachment, cell migration, cell reorganization, and tissue remodeling in response to endothelial damage [89]. YKL-40 acts as an inflammatory factor in various forms of acute and chronic inflammation and is involved in the pathogenesis of several diseases [88,89]. In addition, in kidney diseases, it associates with disease progression and risk of death [90]. YKL-40 levels have been detected to predict the outcome of several infections. YKL-40 level has been found to be connected with the severity of community-acquired pneumonia and outcome of pneumococcal bacteremia, as well as survival in sepsis [91,92,93].

In PUUV infection, plasma YKL-40 levels are elevated during the acute phase [94]. Plasma YKL-40 level associates with the length of hospital stay, which reflects the overall severity of the infection. In addition, also in terms of inflammation and AKI, YKL-40 predicts the severity of the disease caused by PUUV [94]. Furthermore, plasma YKL-40 levels remain elevated during the acute phase of PUUV infection as a sign of undergoing inflammation [94]. However, the YKL-40 level does not correlate with the signs of vascular leakage or with thrombocytopenia [94]. Thus, YKL-40 does not seem to be a major factor in the development of the vascular leakage observed in PUUV infection.

### 2.9. Galectin-3 Binding Protein

Galectin-3 binding protein (Gal-3BP), also known as Mac-2 binding protein, is a ubiquitous and multifunctional secreted glycoprotein found in human serum [95], Increased Gal-3BP levels have been reported in various infections and in several types of cancer [95]. High serum concentrations of Gal-3BP have been detected in both acute and chronic viral infections, e.g., HIV, hepatitis C, Epstein–Barr, and dengue virus infections, and it has also been shown to have predictive value for the severity or progression of the infection [96,97,98,99]. Furthermore, increased levels of Gal-3BP have been detected in the circulation of patients with blood culture-positive bacterial infections [96].

We have detected high levels of Gal-3BP in the acute-stage HFRS induced by PUUV [100]. Furthermore, the levels correlate with disease severity, i.e., variables reflecting fluid retention and the length of hospital stay [100]. Interestingly, the Gal-3BP level also correlates with increased complement activation [100]. These results suggest that Gal-3BP could possess antiviral action by triggering an innate immune response via activation of the complement system [100].

### 2.10. GATA-3

Type 2 cytokine transcription factor (GATA-3) is a necessary transcription factor for the generation of type 2 T cells, and it plays multiple vital roles in the hematopoiesis of many cell lineages [101]. In addition to type 2 T cells, the GATA-3 transcription factor is expressed by distal renal tubular and collecting duct cells [102]. Elevated GATA-3 mRNA levels in circulating mononuclear cells have been reported in patients with minimal change nephrotic syndrome [103].

In acute PUUV infection, urinary sediment GATA-3 mRNA expression is increased when compared with the convalescent phase [104]. Furthermore, urinary sediment GATA-3 level associates with the degree of AKI and acts as an independent risk factor for severe AKI [104]. This may reflect injury in distal tubular or collecting duct cells in the PUUV-infected kidneys [104].

### 2.11. Neutrophil Gelatinase-Associated Lipocalin

Neutrophil gelatinase-associated lipocalin (NGAL) is a ubiquitous glycoprotein originally isolated from human neutrophils [105]. It is also expressed in kidney, liver, and epithelial cells under certain conditions [105]. Inflammation, infection, and kidney injury can upregulate the synthesis of NGAL [105]. Elevated NGAL in the urine or plasma has been recognized as a biomarker of AKI, and the NGAL level is a useful early predictor of AKI across a range of clinical settings [106]. It has been shown to have both diagnostic and prognostic value for AKI [106].

In a German study, urinary NGAL was found to be a good predictor for the severity of AKI due to PUUV infection [107]. NGAL, determined at hospital admission, predicted the severity of AKI and was associated with the length of hospital stay [107]. Therefore, NGAL could help in the initial risk stratification of PUUV patients at hospital admission [107].

### 2.12. Procalcitonin

Procalcitonin (PCT) is an increasingly used biomarker for the early detection of systemic bacterial infections. In healthy individuals, PCT is cleaved from pre-procalcitonin, synthesized by thyroid C cells, and later broken down to form calcitonin [108]. Normally, under physiological conditions, serum PCT levels are very low, but the synthesis of PCT can be rapidly increased as a result of endotoxins or cytokines [108]. The increase in PCT levels is more prominent in bacterial infections than in viral infections or non-infectious inflammatory states, and thus, PCT is used in distinguishing bacterial infections from viral infections or other inflammatory conditions [108]. It is also useful in guiding the treatment of bacterial infections and in decision making of discontinuation of antibiotics [108].

PCT was assessed in a German study in PUUV-infected patients [109]. An elevation of PCT was common, but increased PCT did not associate with a more severe disease, including thrombocytopenia or AKI [109]. Thus, PCT does not seem to be a useful biomarker when assessing the severity of the viral infection caused by PUUV.

## 3. Concluding Remarks

PUUV hantavirus causes usually a mild HFRS. However, the severity of the disease can vary greatly. Host genetics are known to influence the clinical picture. Host immune or inflammatory mechanisms together with virus characteristics and viral load probably have an effect on the development of the clinical disease. Various biomarkers have been studied in an attempt to assess or predict the severity of the infection. Here, we have not reviewed other factors than biomarkers that have shown value in assessing the severity of the infection, e.g., urine analysis. Nevertheless, the amount of proteinuria, hematuria, and glucosuria in a urine dipstick test analysis does have predictive value for the severity of the disease in addition to the various biomarkers [110,111,112].

**Figure 1:**
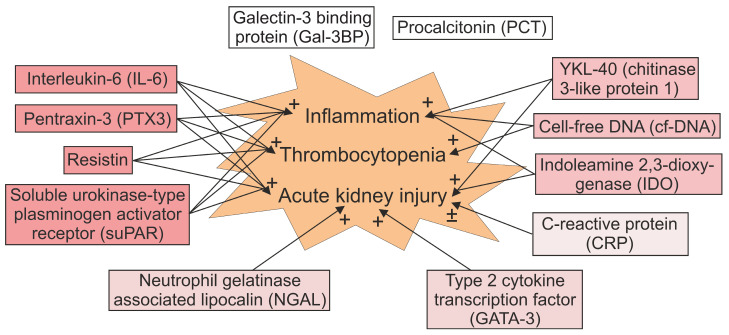
Association between biomarkers and clinical features of Puumala hantavirus infection. See text for detailed explanations. The levels of galectin-3 binding protein (Gal-3BP) correlate with variables reflecting fluid retention and the length of hospital stay [100]. Elevated procalcitonin (PCT) is common in acute infection but does not associate with a more severe disease [109].

Several immunoinflammatory markers have been shown to perform as indicators of the overall severity or some specific characteristics of PUUV infection. Table 1 summarizes the findings about the biomarkers regarding the length of hospital stay, and the severity of AKI, thrombocytopenia, and leukocytosis. The associations between the biomarkers and different clinical features of PUUV infection are presented in Figure 1. However, as the exact pathogenetic mechanisms of the disease have not been revealed, it is indistinguishable to what extent the upregulation of a particular biomarker reflects the immunological activation targeting at eliminating the virus and to what extent it is due to the response to tissue damage. Although the production of the various immunoinflammatory mediators is often co-regulated, different biomarkers probably reflect different immunopathogenic characteristics of the disease. Finally, growing knowledge about the biomarkers have also clarified the pathogenesis of HFRS induced by PUUV.

## Figures and Tables

**Table 1:** The associations of different elevated biomarkers with the length of hospital stay, acute kidney injury, thrombocytopenia, and leukocytosis in Puumala hantavirus infection.

Biomarker	Length of Hospital Stay	AKI	Thrombocytopenia	Leukocytosis	Reference
IL-6	Yes	Yes	Yes	Yes	[33]
CRP	No	*	No	No	[33,43]
PTX-3	Yes	Yes	Yes	Yes	[54]
IDO	Yes	Yes	No	Yes	[63]
cf-DNA	Yes	No	Yes	Yes	[67]
suPAR	Yes	Yes	Yes	Yes	[76,78]
Resistin	Yes	Yes	Yes	Yes	[87]
YKL-40	Yes	Yes	No	Yes	[94]
Gal-3BP	Yes	No	No	No	[100]
GATA-3	NS	Yes	NS	NS	[104]
NGAL	Yes	Yes	NS	NS	[107]
PCT	No	No	No	No	[109]

AKI = acute kidney injury, * = contradictory results, NS = not studied, IL-6 = interleukin-6, CRP = C-reactive protein, PTX-3 = pentraxin-3, IDO = indoleamine 2,3-dioxygenase, cf-DNA = cell-free DNA, suPAR = soluble urokinase-type plasminogen activator, Gal-3BP = galectin-3 binding protein, NGAL = neutrophil gelatinase-associated lipocalin, PCT = procalcitonin.

## Data Availability

Not applicable.
